# Polypeptone Induces Dramatic Cell Lysis in *ura4* Deletion Mutants of Fission Yeast

**DOI:** 10.1371/journal.pone.0059887

**Published:** 2013-03-21

**Authors:** Yuzy Matsuo, Kouhei Nishino, Kouhei Mizuno, Takashi Akihiro, Takashi Toda, Yasuhiro Matsuo, Tomohiro Kaino, Makoto Kawamukai

**Affiliations:** 1 Department of Life Science and Biotechnology, Faculty of Life and Environmental Science, Shimane University, Matsue, Japan; 2 Department of Biological Science, Faculty of Life and Environmental Science, Shimane University, Matsue, Japan; 3 Cell Regulation Laboratory, London Research Institute, Cancer Research UK, London, United Kingdom; St Jude Children’s Research Hospital, United States of America

## Abstract

Polypeptone is widely excluded from *Schizosaccharomyces pombe* growth medium. However, the reasons why polypeptone should be avoided have not been documented. Polypeptone dramatically induced cell lysis in the *ura4* deletion mutant when cells approached the stationary growth phase, and this phenotype was suppressed by supplementation of uracil. To determine the specificity of this cell lysis phenotype, we created deletion mutants of other genes involved in *de novo* biosynthesis of uridine monophosphate (*ura1*, *ura2*, *ura3*, and *ura5*). Cell lysis was not observed in these gene deletion mutants. In addition, concomitant disruption of *ura1*, *ura2*, *ura3*, or *ura5* in the *ura4* deletion mutant suppressed cell lysis, indicating that cell lysis induced by polypeptone is specific to the *ura4* deletion mutant. Furthermore, cell lysis was also suppressed when the gene involved in coenzyme Q biosynthesis was deleted. This is likely because Ura3 requires coenzyme Q for its activity. The *ura4* deletion mutant was sensitive to zymolyase, which mainly degrades (1,3)-beta-D glucan, when grown in the presence of polypeptone, and cell lysis was suppressed by the osmotic stabiliser, sorbitol. Finally, the induction of cell lysis in the *ura4* deletion mutant was due to the accumulation of orotidine-5-monophosphate. Cell wall integrity was dramatically impaired in the *ura4* deletion mutant when grown in the presence of polypeptone. Because *ura4* is widely used as a selection marker in *S. pombe*, caution needs to be taken when evaluating phenotypes of *ura4* mutants.

## Introduction

The fission yeast, *Schizosaccharomyces pombe*, is a eukaryotic model organism used to study a wide range of molecular and cellular biological processes, including cell cycle regulation, signal transduction, cell polarity control, and chromatin structure [1]–[3]. *S. pombe* is also used to study the mechanisms responsible for controlling cell wall synthesis and cellular morphogenesis [4].

The composition of the media on which *S. pombe* is grown is an important factor that needs to be considered during phenotypic analysis. *S. pombe* is commonly grown on the minimum medium, EMM, and the rich medium, YE [5]. The sensitivity of *S. pombe* to drugs or temperature often depends on the growth media used; for example, *S. pombe* is less sensitive to G418 when grown on EMM than when grown on YE. Growth media commonly used for *S. cerevisiae*, such as YPD that contains polypeptone, and SD that contains a nitrogen base, are not widely used to grow *S. pombe*. This is because many researchers have observed that *S. pombe* grown on these media can exhibit unexpected and unwanted alterations in the phenotypes of interest. Although polypeptone in YPD media is generally considered to be responsible for these effects, the reasons for this have not been thoroughly investigated.

The *leu1*, *ade6*, and *ura4* genes are genetic markers commonly used for selection in *S. pombe*. However, these selectable marker genes can affect the phenotype of interest; for example, specific amino acids affect sexual differentiation [6].

We observed dramatic cell lysis of the *ura4* deletion mutant grown in the presence of polypeptone. This was specific to the *ura4* gene and was not observed in the other uracil auxotrophs, *ura1*, *ura2*, *ura3*, and *ura5*. Cell lysis was also observed when the *ura4* strain was grown on YE media, although to a lesser extent. These results indicate that caution must be taken when interpreting phenotypes of *ura4* deletion mutants of *S. pombe*. Our analysis indicates that *ura4* specifically, rather than other genes involved in *de novo* biosynthesis of uridine monophosphate (UMP), affects cell wall integrity.

## Materials and Methods

### Strains, Media, and Genetic Manipulation

The *S. pombe*, *S. japonicas*, *and S. cerevisiae* strains used in this study are listed in Table 1. Standard yeast culture media and genetic manipulations were used [5]. *S. pombe* strains were grown in complete YES medium (0.5% yeast extract, 3% glucose, 225 mg/L each of adenine, leucine, uracil, histidine, and lysine hydrochloride), in YPD medium (1.0% yeast extract, 2% glucose, and 2% polypeptone), or in EMM medium (0.3% potassium hydrogen phthalate, 0.56% sodium phosphate, 0.5% ammonium chloride, 2% glucose, vitamins, minerals, and salts). The appropriate auxotrophic supplements were added as necessary (75 mg/L of adenine, leucine, uracil, histidine, lysine, or cysteine). YE medium (0.5% yeast extract and 3% glucose), YTD medium (0.5% yeast extract, 3% glucose, and 2% tryptone) and YCD medium (0.5% yeast extract, 3% glucose, and 2% casamino acids) were also used. SPA medium was used to induce sporulation. Phloxin B was added to a final concentration of 5 mg/L. G418 disulphate (Sigma Co. Ltd), hygromycin B (Wako Co. Ltd), and 5*-*Fluoroorotic acid (5-FOA) (Wako Co. Ltd) were used in solid YES plates at a concentration of 100 mg/L, 150 mg/L, and 1 g/L, respectively. Calcofluor white M2R (Sigma chemical) was used to test the sensitivity of strains on YES or YPD plates.

**Table 1:** *S. pombe*, *S. japonicus* and *S. cerevisiae* strains used in this study.

Schizosaccharomyces pombe		
Strain	Genotype	Source
L972	*h^−^*	Lab stock
L975	*h^+^*	Lab stock
L968	*h^90^*	C. Shimoda
PR109	*h^−^ leu1-32 ura4-D18*	P. Russel
PR110	*h^+^ leu1-32 ura4-D18*	P. Russel
SP870	*h^90^ leu1-32 ura4-D18 ade6-M216*	[13]
TP4-1D	*h^+^ leu1-32 ura4-D18 his2 ade6-M216*	[14]
TP4-5A	*h^−^ leu1-32 ura4-D18 ade6-M210*	[14]
RM1	*h^+^ leu1-32 ura4-D18* Δ*coq7::kanMX6*	[23]
UMP31	*h^−^ Δura4::kanMX6*	This study
UMP32	*h^−^ Δura4::hphMX6*	This study
UMP33	*h^+^ Δura4::hphMX6*	This study
UMP34	*h^−^ Δura1::kanMX6*	This study
UMP35	*h^−^ Δura2::kanMX6*	This study
UMP36	*h^−^ Δura3::kanMX6*	This study
UMP37	*h^−^ Δura5::kanMX6*	This study
UMP38	*h^−^ Δcoq8::kanMX6*	This study
UMP39	*h^−^ Δura4::hphMX6 Δura1::kanMX6*	This study
UMP40	*h^−^ Δura4::hphMX6 Δura2::kanMX6*	This study
UMP41	*h^−^ Δura4::hphMX6 Δura3::kanMX6*	This study
UMP42	*h^−^ Δura4::hphMX6 Δura5::kanMX6*	This study
UMP43	*h^−^ Δura4::hphMX6 Δcoq8::kanMX6*	This study
UMP44	*h^+^ Δura4::hphMX6 esc1*	This study
UMP45	*h^−^ esc1 leu1-32*	This study
UMP46	*h^+^ Δura1::kanMX6*	This study
*Schizosaccharomyces japonicus*		
NIG2028	*h^−^*	H. Niki
NIG5091	*h^−^ ura4-D3*	H. Niki
*Saccharomyces cerevisiae*		
SP1	*MATa leu2 ura3 trp1 his3 ade8 can1*	Lab stock
W303A	*MATa ade2-1 trp1-1 leu2-3 112 his1-11 ura3 can1-100*	Lab stock

*Escherichia coli* DH5α was the host strain for all plasmid manipulations, and was grown in LB medium (1% bactotryptone, 0.5% yeast extract, and 1% NaCl, pH 7.0).

### DNA Manipulations and Plasmids

Standard molecular biology techniques were followed as previously described [7]. Restriction enzymes (*Bam*HI and *Sal*I) were used according to the supplier’s recommendation (TOYOBO). Nucleotide sequences were determined by the dideoxynucleotide chain-termination method using an Applied Biosystems 3500 Genetic Analyzer. The plasmids pREP1-*ura1*, pREP1-*ura4*, pREP1-*URA3*, pREP2-*ura2*, pREP2-*ura3*, and pREP2-*ura5* were constructed using the pREP1 or pREP2 [8] vectors and the primers listed in Table S1 with the gap repair cloning method as previously described [9]. The plasmids pFA6a-kanMX6 [10] and pCR2.1-hphMX6 [11] were used as templates to amplify DNA fragments to construct the gene deletion mutants. A pAL-SK-based genomic DNA library (provided from Dr. T. Nakamura) was used to screen the responsible genes of the revertants derived from the *ura4* deletion mutant.

### Gene Disruption and Marker Switch

Chromosomal genes were disrupted using PCR generated fragments [10]. The 1.6 kb kanMX6 module was amplified with flanking homology sequences corresponding to the 5′ and 3′ ends of the target genes. G418-resistant colonies were selected on YES plates containing G418. Correct disruption of the gene of interest was verified by colony PCR. One-step marker switch from kanMX6 to hphMX6 was performed as previously described [11].

### Zymolyase Assay to Assess Cell Wall Integrity

Fission yeast cells were pre-grown at 30°C in YES media and were then grown at 30°C to mid-log phase in YPD media. Cells were harvested by centrifugation and washed with water and TE (10 mM Tris-HCl, pH 8.0, and 1 mM EDTA). Cells were re-suspended in TE and incubated at 30°C for 180 min in the absence or presence of 0.1 mg/ml Zymolyase20T (Seikagaku Kogyo). The degree of cell lysis was evaluated by measuring the optical density at 595 nm.

### Alkaline Phosphatase Assay to Assess Cell Lysis

Cells were grown on YES plates for 3 days at 30°C and were re-suspended in water to a density of 10^7^ cells/ml. Cell suspensions were serially diluted (1∶10) and plated on YES or YPD plates and incubated for 3 days at 30°C. For the alkaline phosphatase assay, each plate was overlaid for 10, 30, and 60 min with a phosphatase assay solution containing 0.05 M glycine-NaOH (pH 9.8), 1% agar, and 2.5 mg/ml 5-bromo-4-chloro-3-indorylphosphate (BCIP).

### Cell Cultivation and Sample Preparation for Liquid Chromatography-mass Spectrometry (LC-MS) Analysis

Extraction of metabolites from yeast cells was performed as previously described [12]. The *S. pombe* wild-type (WT) (L972) and Δ*ura4* (UMP31) strains, and the SP1 and W303A *ura3* mutant *S. cerevisiae* strains, were grown in YPD media. Cells (4×10^8^) were collected by centrifugation, washed with H_2_O, immediately quenched in methanol at −80°C, and then collected again by centrifugation. Cells were disrupted using a Multi-Beads Shocker (Yasui Kikai Co.) in 800 µL of 50% methanol. Proteins were removed with an Amicon Ultra 10 kDa cut-off filter (Millipore), and the samples were concentrated by vacuum evaporation. Finally, each sample was re-suspended in 80 µL of 50% acetonitrile and 50% 20 mM ammonium formate. Samples were filtrated with YMC Duo-Filter QDUO 04 (pore size 0.2 µm) and 2 µL was used for injection.

LC-MS data were obtained using a MassLynx system (Waters) coupled to a Xexo-TQS mass spectrometer (Waters). LC separation was performed on an ACQUITY UPLC BEH Amide column (Merck SeQuant; 2.1×100 mm, 1.7 µm particle size). The mobile phase was 20 mM ammonium formate, pH 3.1 (buffer A) and acetonitrile buffer (buffer B). The chromatographic conditions were 30% buffer A and 70% buffer B at 2 min, which increased immediately to 50% buffer A at 3 min. This condition was maintained for 6 min and the initial condition was restored after 9 min. The flow rate was 0.4 ml/min. Matrix assisted laser desorption/ionisation-time of flight-of-mass spectrometry (MALDI-TOF MS) (SYNAPT G2-S; Waters Corps.) was used to determine the precise molecular masses of compounds.

## Results

### *S. pombe ura4* Deletion Mutants Undergo Dramatic Cell Lysis when Grown in YPD Media

YPD media is widely used to grow *S. cerevisiae*, but not *S. pombe*. Although the exact reasons why *S. pombe* should not be grown in YPD media have not been reported, many researchers have observed that polypeptone in YPD media has undesirable effects on *S. pombe* growth. The PR110 strain (*h^+^ leu1-32 ura4-D18*) lysed upon reaching late-log phase in YPD liquid medium, whereas the WT strain L972 did not (data not shown).

**Figure 1:**
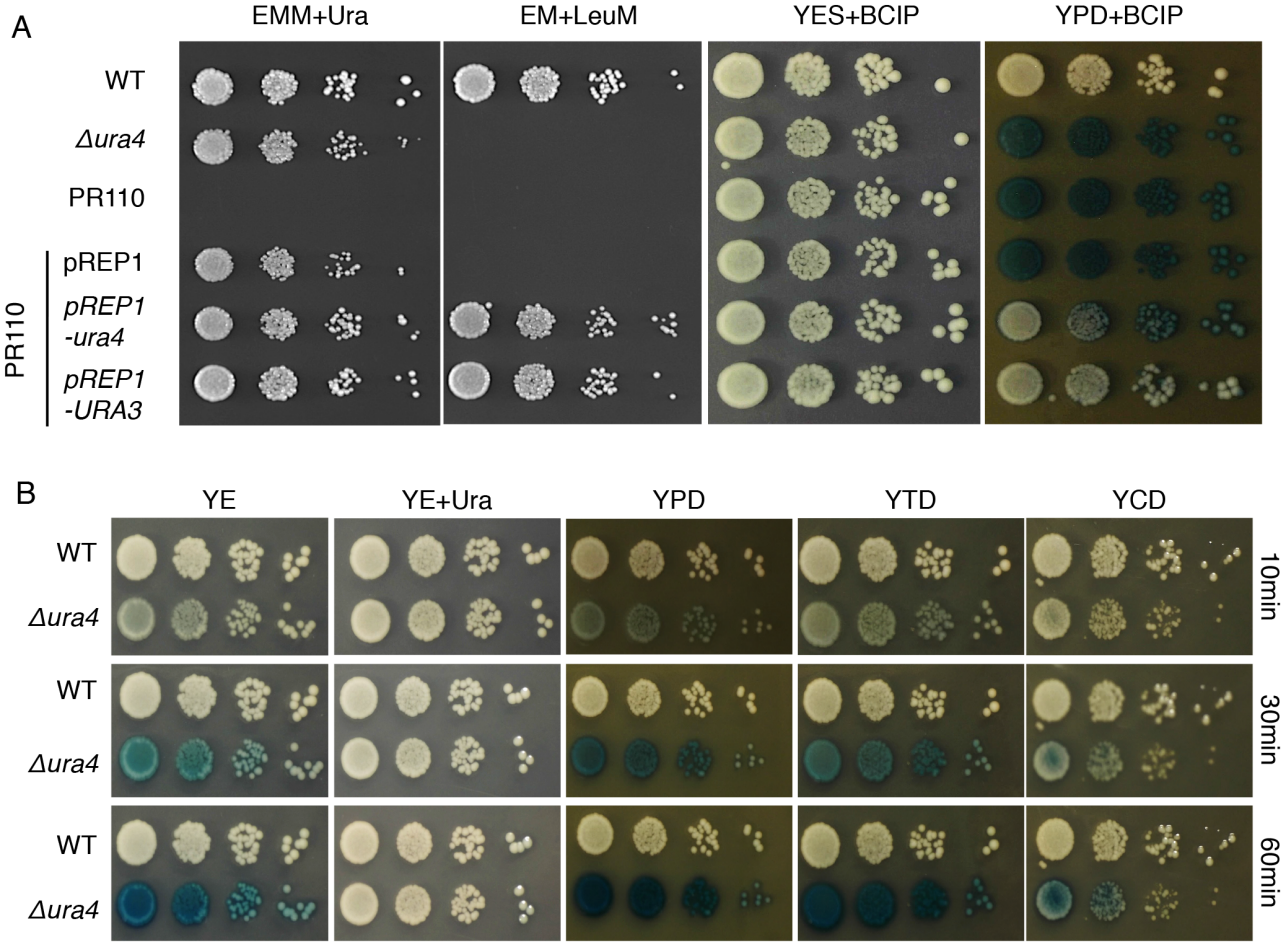
Lysis of Δ*ura4* cells by polypeptone. (A) L972 (WT), UMP31 (Δ*ura4*), PR110 strains, and PR110 containing empty vector (pREP1), pREP1-*ura4* or pREP1-*URA3* were grown on the indicted plates for 3 days at 30°C. Cells were serial diluted by 10-fold. For the alkaline phosphatase assay, YPD plates were overlaid for 10 min with a phosphatase assay solution containing 0.05 M glycine-NaOH (pH 9.8), 1% agar, and 2.5 mg/ml of BCIP. (B) L972 (WT) and UMP31 (Δ*ura4*) strains were serially diluted by 10-fold and spotted onto the indicated plates and incubated for 3 days at 30°C. For the alkaline phosphatase assay, each plate was overlaid with the phosphatase assay solution for the indicated time as described in (A).

To determine whether the absence of leucine or uracil in the PR110 strain is responsible for this, we separated the two markers by crossing PR110 with L972 and isolated four siblings. Only strains that lacked uracil lysed in YPD media (Fig. S1). This phenotype was visualised in the form of blue colonies by staining with BCIP, which is an artificial chromogenic substrate that is converted to blue dye by endogenous alkaline phosphatase (Fig. 1A). The blue color became clear in Δ*ura4* cells after incubation with BCIP for 30 min. Cell lysis of PR110 was suppressed in YES medium that contained uracil, or by introducing pREP2 containing the *ura4* gene (pREP2-*ura4*) or the *S. cerevisiae URA3* gene (pREP2-*URA3*), which is a *ura4* ortholog. This indicates that uracil auxotrophy by deletion of *ura4* is responsible for the cell lysis phenotype. Δ*ura4* cells grown on YE medium also underwent cell lysis, although to a lesser extent, and this was enhanced by supplementation of polypeptone or tryptone, but not casamino acids (Fig. 1B). This suggests that components of polypeptone or tryptone, apart from amino acids, enhance cell lysis. Other *ura4* deletion mutants, including the SP870 [13], TP4-1D, and TP4-5A strains [14], also underwent cell lysis (data not shown).

**Figure 2:**
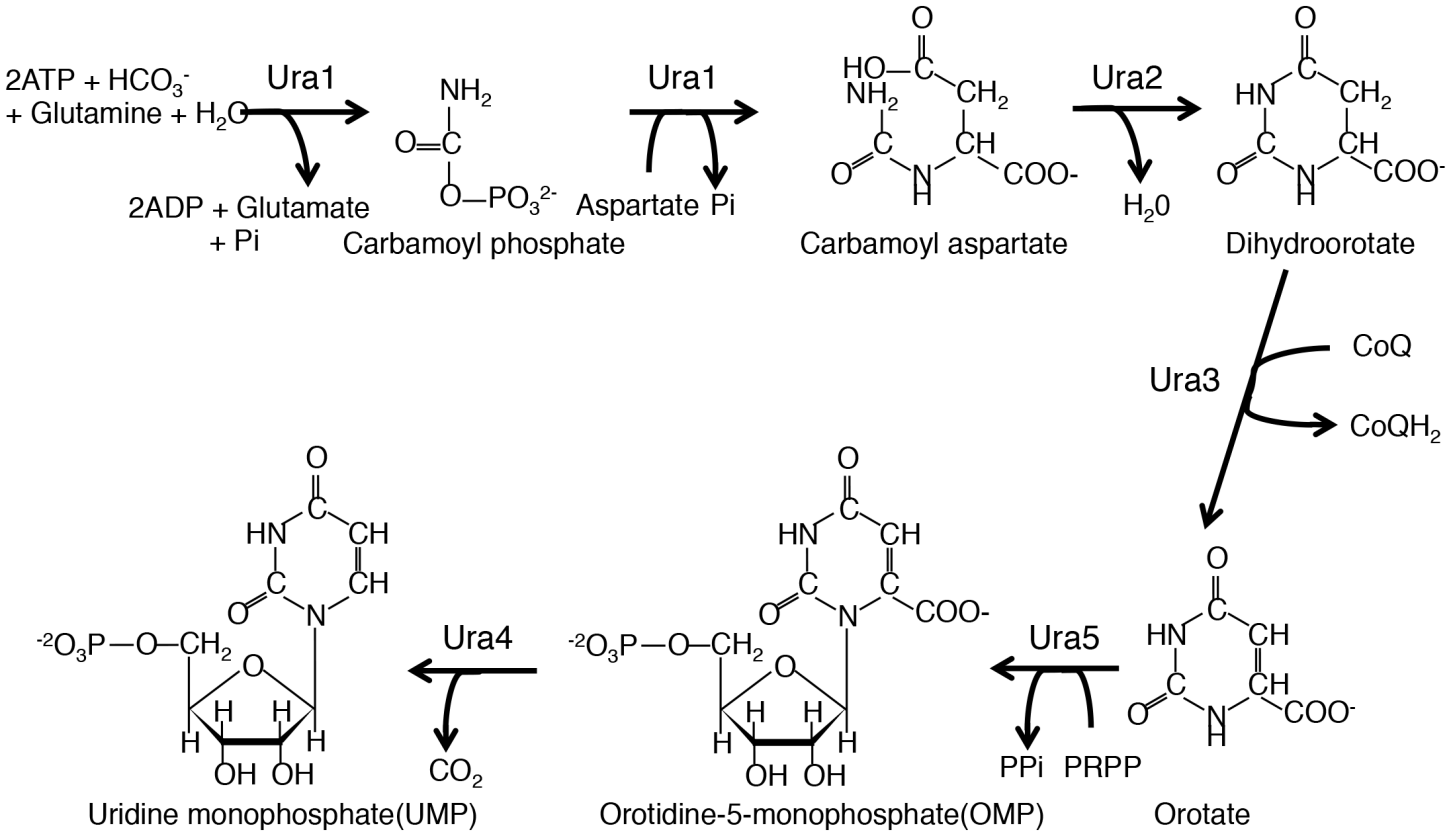
*De novo* UMP synthesis in *S. pombe* involves six steps and five enzymes. The *de novo* synthesis of UMP in *S. pombe* is outlined. Ura1 is a bi-functional enzyme consisting of carbamoyl phosphate synthetase I and aspartate transcarbamoylase, Ura2 is dihydroorotase, Ura3 is dihydroorotate dehydrogenase that requires quinone as a cofactor and localizes in mitochondria, Ura5 is orotate phosphoribosyltransferase, and Ura4 is OMP decarboxylase.

**Figure 3:**
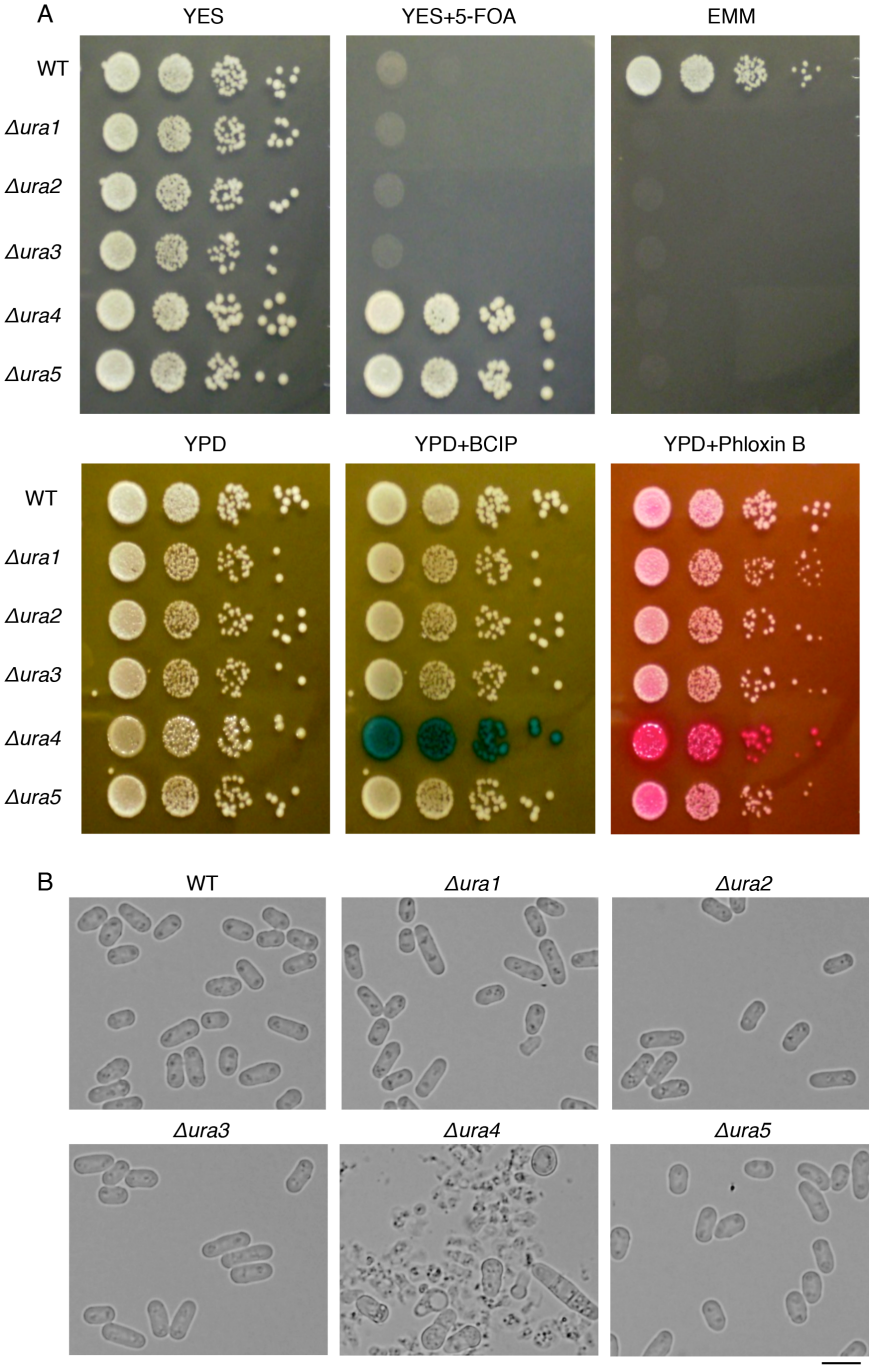
Cell lysis caused by polypeptone specifically occurs in the *ura4* deletion mutant. (A) Cell cultures of L972 (WT), UMP34 (Δ*ura1*), UMP35 (Δ*ura2*), UMP36 (Δ*ura3*), UMP31 (Δ*ura4*), and UMP37 (Δ*ura5*) strains were serially diluted by 10-fold, plated on the indicated plates and incubated for 3 days at 30°C. For the alkaline phosphatase assay, BCIP was used as described in Fig. 1. (B) Cellular morphologies of the indicated strains after being grown on YPD plates for 3 days at 30°C. Scale bar: 10 µm.

We generated deletion mutants of genes involved in *de novo* metabolism of UMP to further explore the relationship between uracil auxotrophy and cell lysis. The *ura4* gene encodes orotidine-5-monophosphate (OMP) decarboxylase that mediates the formation of UMP from OMP. Four other genes (*ura1*, *ura2*, *ura3*, and *ura5*) are also involved in *de novo* synthesis of UMP in *S. pombe* (Fig. 2) [15]–[17]. We observed the phenotypes of strains in which these genes were disrupted. All five *ura* deletion mutants showed uracil auxotrophy, and the *ura4* and *ura5* deletion strains were resistant to 5-FOA as expected (Fig. 3A). When the *ura4* deletion strain was grown on YPD plates in the presence of phloxin B, it formed darker red colonies than the other strains, and it formed dark blue colonies when grown in the presence of BCIP (Fig. 3A), indicating that cell lysis occurred specifically in the *ura4* deletion mutant. Addition of uracil in YPD medium suppressed the cell lysis in the *ura4* deletion strain (Fig. S2). Microscopic observation also indicated that cells of the *ura4* deletion mutant lysed, whereas those of the other four mutants were normal (Fig. 3B).

### The *ura4* Deletion Mutant has a Defect in Cell Wall Integrity when Grown in YPD Media

**Figure 4:**
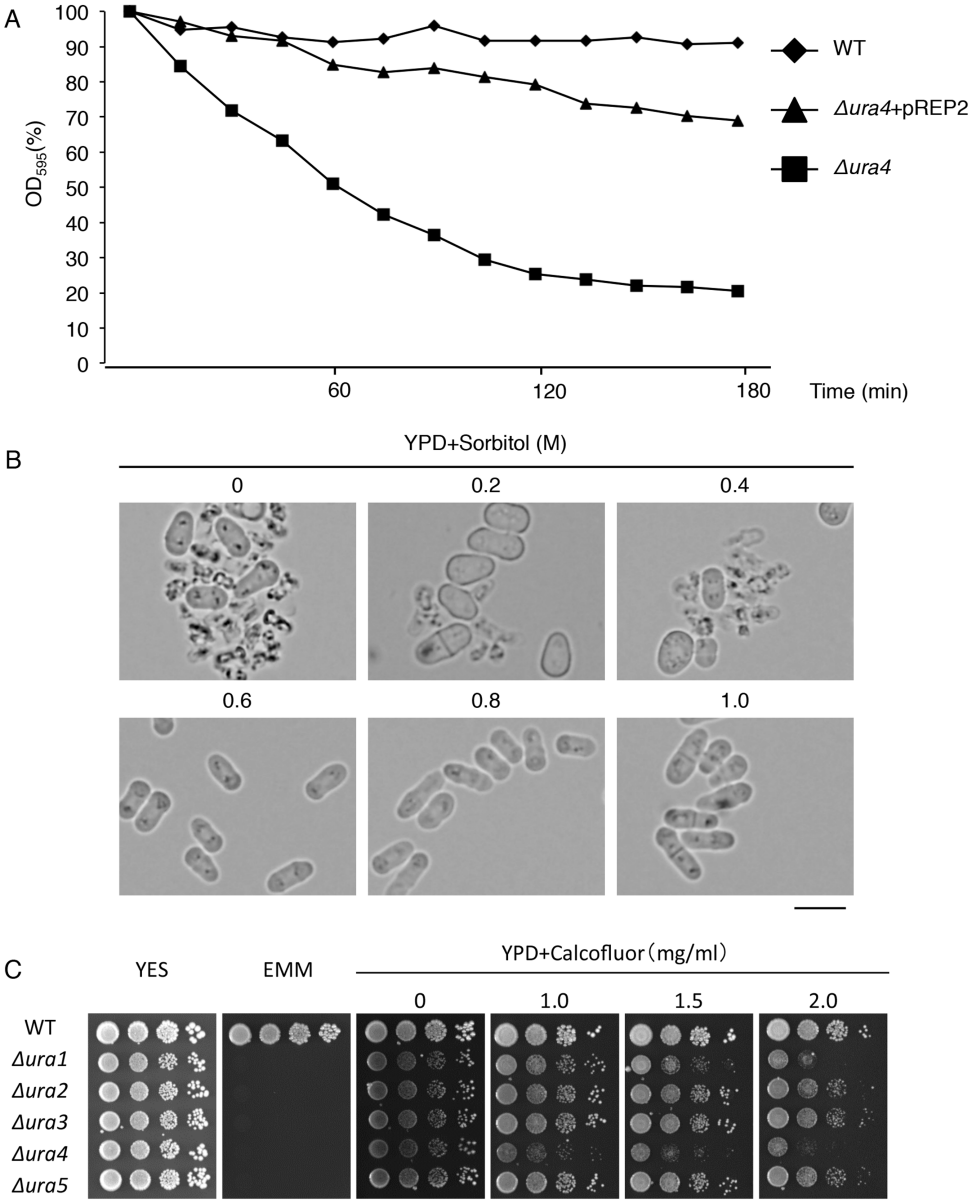
Cell wall integrity is impaired in the *ura4* deletion mutant when grown in the presence of polypeptone. (A) L972 (WT) and *Δura4* strains and *Δura4* containing pREP2 were grown at 30°C in YPD media and treated with 0.1 mg/ml Zymolyase20T. The degree of cell lysis was evaluated by measuring the optical density at 595 nm (OD_595nm_). The OD_595nm_ at various time-points relative to the value at 0 min is indicated. (B) The morphology of UMP31 (*Δura4*) cells grown in YPD media in the presence of various concentrations of sorbitol. Scale bar: 10 µm (C) The indicated strains were serially diluted 10-fold and grown on YES, EMM or YPD media containing calcofluor white for 4 days at 30°C.

When the *ura4* deletion mutant was grown in YPD media, cells had a round or a tadpole-like morphology before cell lysis began (Fig. 3B). Because this morphology is often found in mutants that a defect in cell wall integrity [18], we suspected that the morphology defect and cell lysis of the *ura4* deletion mutant grown in YPD media was due to a defect in cell wall integrity. To test this, we examined the sensitivity of the *ura4* deletion mutant to ß-glucanase (zymolyase20T), which breaks down cell walls. The *ura4* deletion mutant was more sensitive to zymolyase than the WT strain and the *ura4* deletion mutant containing pREP2 (Fig. 4A). We then tested whether the osmotic stabiliser, sorbitol, could rescue the cell lysis phenotype of the *ura4* deletion mutant. Cell lysis was suppressed when sorbitol was added at a concentration of 0.6 M or higher (Fig. 4B). Consistent with these observations, the *ura4* deletion mutant was sensitive to calcofluor white, whereas the *ura2*, *ura3*, and *ura5* deletion mutants were not (Fig. 4C). Calcofluor white inhibits the growth of *S. pombe* mutants that have defects in the integrity of cell walls [19]. These results suggest that the morphological defects and cell lysis of the *ura4* gene deletion mutant grown in YPD media is due to a defect in cell wall integrity. The *ura1* deletion mutant was also sensitive to calcofluor white (Fig. 4C), but we do not have clear explanation of this phenotype.

### Disruption of Genes Upstream of *ura4* in UMP Synthesis Suppresses the Cell Lysis Phenotype of the *ura4* Deletion Mutant

**Figure 5:**
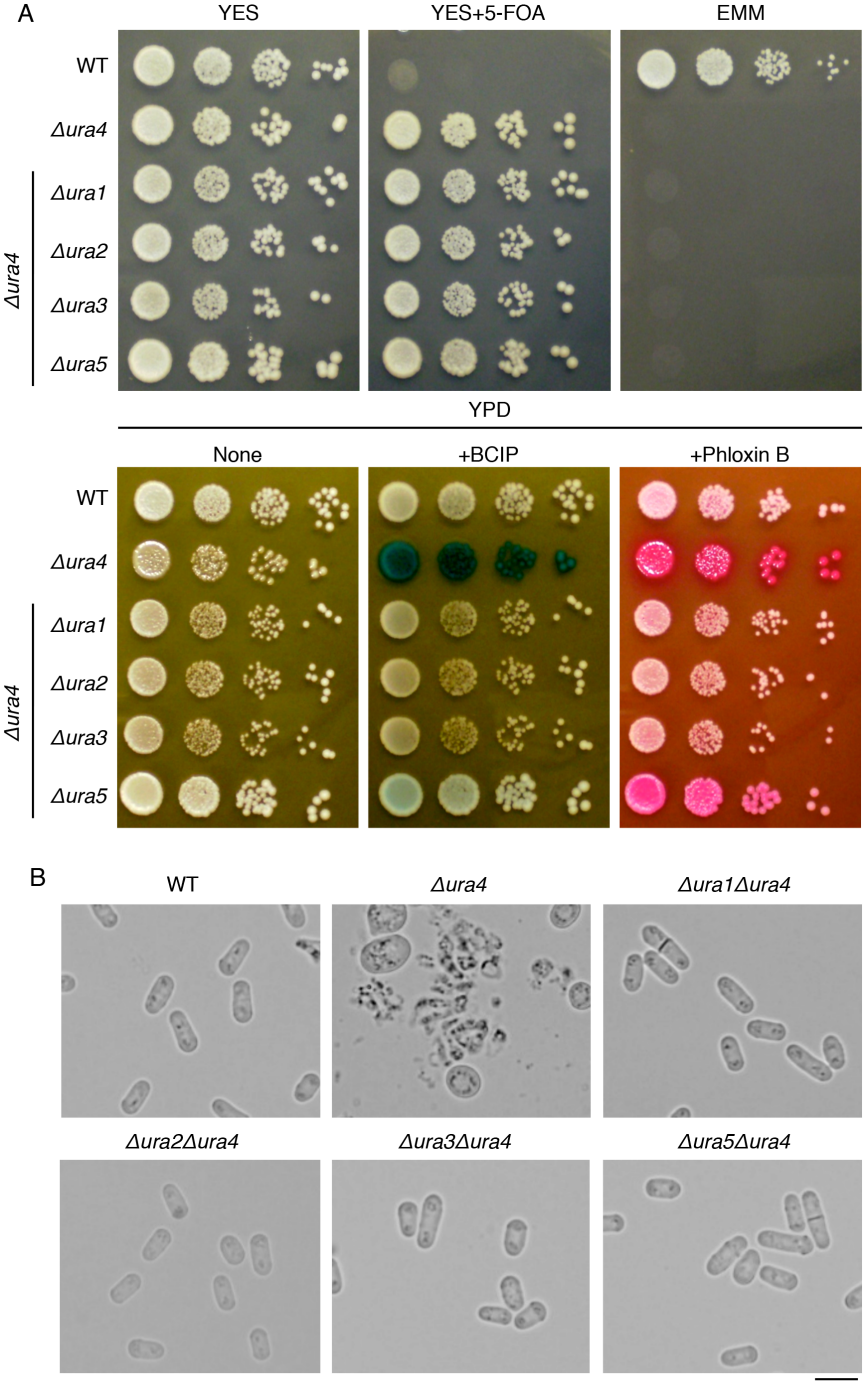
Concomitant disruption of *ura1*, *ura2*, *ura3*, or *ura5* in the *ura4* deletion mutant suppresses cell lysis in the presence of polypeptone. (A) Serially diluted cells (10-fold) of L972 (WT), UMP31 (Δ*ura4*), UMP39 (Δ*ura1*Δ*ura4*), UMP40 (Δ*ura2*Δ*ura4*), UMP41 (Δ*ura3*Δ*ura4*), and UMP42 (Δ*ura5*Δ*ura4*) strains were grown on the indicated plates and incubated for 3 days at 30°C. For the alkaline phosphatase assay, BCIP was used as described in Fig. 1. (B) Cellular morphologies of the indicated strains after being grown on YPD plates for 3 days at 30°C. Scale bar: 10 µm.

Ura4 catalyses the last step of UMP synthesis, and Ura1, Ura2, Ura3, and Ura5 catalyse the reactions upstream of Ura4 (Fig. 2) [17]. We created double deletion mutants to determine whether concomitant disruption of *ura1*, *ura2*, *ura3*, or *ura5* in the *ura4* deletion mutant affected the cell lysis phenotype. The cell lysis of the *ura4* deletion mutant was markedly suppressed by concomitant disruption of *ura1*, *ura2*, *ura3*, or *ura5* as indicated by BCIP and phloxin B staining (Fig. 5A) and cellular morphology (Fig. 5B). Thus, *ura4* was predominantly responsible for the cell lysis induced by polypeptone.

**Figure 6:**
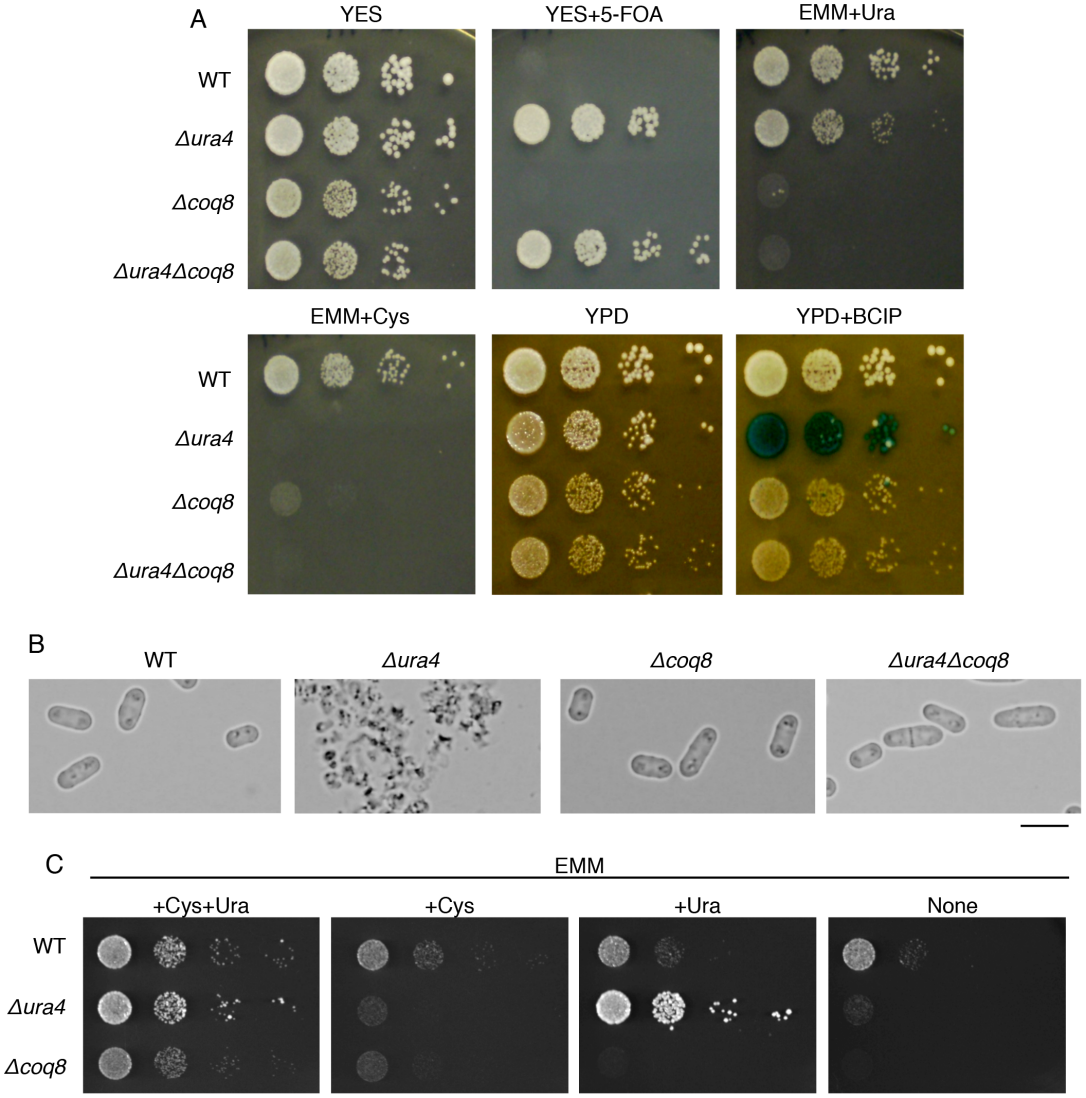
Deletion of *coq8* suppresses the cell lysis phenotype of the *ura4* deletion mutant. (A) Serially diluted cells (10-fold) of L972 (WT), UMP31 (Δ*ura4*), UMP38 (Δ*coq8*), and UMP43 (Δ*coq8*Δ*ura4*) strains were grown on the indicated plates and incubated for 3 days at 30°C. For the alkaline phosphatase assay, BCIP was used as described in Fig. 1. (B) Cellular morphologies of the indicated strains after being grown on YPD plates for 3 days at 30°C. Scale bar: 10 µm. (C) Serially diluted cells (10-fold) of L972 (WT), UMP31 (Δ*ura4*), and UMP38 (Δ*coq8*) strains were grown on the indicated plates and incubated for 3 days at 30°C.

In *S. pombe*, Ura3 localizes in the inner membrane of mitochondria and requires quinone for its activity [20]. Because coenzyme Q is the only quinone that exists in the mitochondria, we hypothesised that genes involved in the biosynthesis of coenzyme Q might be required for *de novo* biosynthesis of UMP. To test this possibility, we disrupted the *coq*8 gene, which is required for coenzyme Q synthesis [21], [22]. Disruption of *coq8* suppressed the cell lysis phenotype of the *ura4* deletion mutant (Fig. 6A and B). The *coq8* deletion mutant showed uracil auxotrophy similar to gene deletion mutants involved in *de novo* metabolism of UMP (Fig. 6C). This suggests that suppression of the cell lysis phenotype of the *ura4* deletion mutant by deletion of *coq8* is mediated by UMP metabolism. Cysteine was added to the medium, as this is required to grow the *coq* deletion mutant on minimum medium [14], [21], [22]. Another *coq* mutant, RM1 (*coq7*) [23], also suppressed the cell lysis phenotype of the *ura4* deletion mutant (data not shown).

### Search for Genes that Contribute to the Cell Lysis Phenotype of the *ura4* Gene Deletion Mutant

To identify genes that might contribute to the cell lysis phenotype, we attempted to isolate multi-copy suppressors that suppressed the cell lysis of the *ura4* deletion mutant when grown on YPD media. PR110 was transformed with a pAL-SK-based genomic DNA library and transformants were selected on EMM plates. All transformants (approximately 50,000 colonies) were replica-plated onto YPD plates containing phloxin B and incubated at 30°C for 2 days. Four colonies were identified that were more weakly stained than the *ura4* deletion mutant by phoxin B, indicating that cell lysis was suppressed. Plasmids were recovered from these colonies and the plasmid genes were identified. All plasmids contained the *ura4* gene (data not shown). Thus, no multi-copy suppressor genes were identified at least in our screening. This confirmed that *ura4* is fully responsible for the cell lysis of the *ura4* deletion mutant when grown in the presence of polypeptone.

To gain further insight into the mechanism by which cell lysis is induced in the *ura4* deletion strain, we isolated spontaneous revertants that suppressed cell lysis of the *ura4* deletion mutant. UMP33 cells (*h^+^* Δ*ura4::hphMX6*) were incubated on a YPD plate containing phloxin B at 30°C for 1 week. Eight independent spontaneous revertants were screened by observing colony color and cell morphology. Tetrad analyses showed that extragenic suppression of revertants was caused by a single genetic locus and that this suppression was recessive. These were designated *esc* (extragenic suppressor of cell lysis) mutants. These revertants were back-crossed with the WT strains L972 and L975 to eliminate secondary mutations. Genetic analysis established that these eight mutant alleles were derived from one genetic locus and these mutants showed uracil auxotrophy. To isolate the responsible genes of the *esc* mutants, the UMP45 (*h^−^ esc1 leu1-32*) strain was transformed with a pAL-SK-based genomic DNA library and selected on EMM plates. All transformants (approximately 50,000 colonies) were replica-plated onto EMM plates without uracil and incubated at 30°C for 5 days. The *ura1* gene was identified by sequencing of the clones. The sequencing of the *ura1* locus of UMP45 further identified an insertion mutation in the *ura1* ORF between bases 135 and 136 (data not shown). These results reconfirmed that disruption of genes upstream of *ura4* in UMP synthesis suppresses the cell lysis phenotype of the *ura4* deletion mutant.

### Accumulation of Precursors of OMP in the *ura4* Deletion Mutant

**Figure 7:**
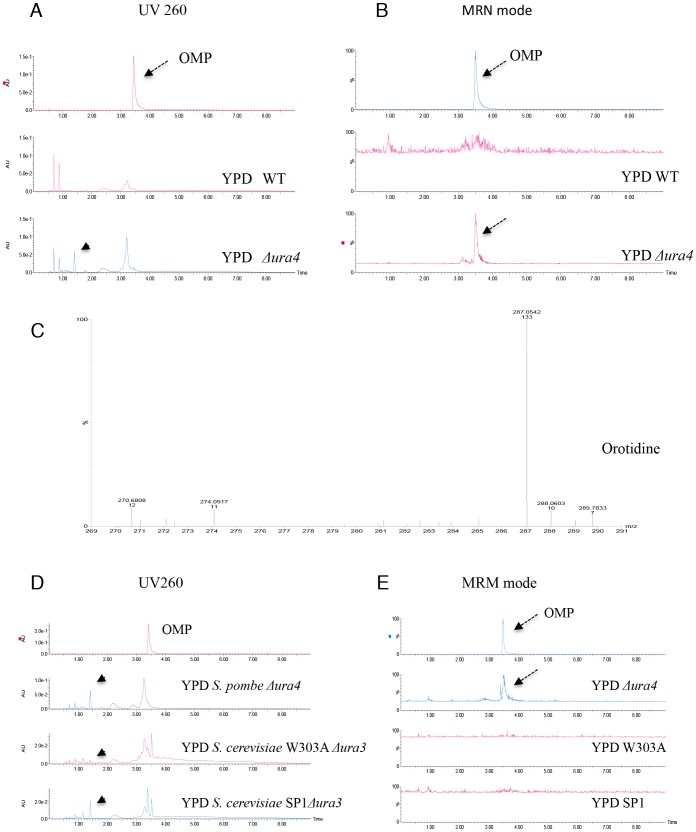
Mass spectrometry analysis of OMP precursors in the *ura4* deletion mutant. (A) HPLC analysis of L972 (WT) and UMP31 (Δ*ura4*) cells grown in YPD media. Metabolites were extracted in 50% methanol from bead-disrupted cells and isolated by a 10 kDa cut-off filter. A peak corresponding to OMP was not observed in the samples, but a specific peak at 1.40 (arrow head) was observed in the UMP31 (Δ*ura4*) sample. (B) A peak at 367 m/z (the size of OMP) that was detected by mass spectrometry in negative ESI mode was fragmented in MRM mode (MS/MS). A peak at 3.51 (arrow) observed in metabolites from UMP31 (Δ*ura4*) coincides well with the peak of OMP. (C) The peak in the UMP31 (Δ*ura4*) sample at 1.40 detected by absorbance at 260 nm was identified as orotidine when analysed by TOF-MS (range 270–290 m/z) in ionization mode. (D) HPLC analysis of *S. pombe* UMP31 (Δ*ura4*) cells, and *S. cerevisiae* SP1 and W303A *ura3* mutant cells grown in YPD media. Peaks corresponding to orotidine (arrow heads) were observed. (E) A peak at 367 m/z was fragmented by MS/MS in negative ESI mode. A peak corresponding to OMP (arrow) was observed in the UMP31 (Δ*ura4*) sample, but not in the *S. cerevisiae ura3* mutants.

Ura4 is thought to catalyse the decarboxylation of OMP to UMP in *S. pombe* based on its amino acid sequence similarity with other orthologs and genetic complementation analyses [17]; however, metabolic analysis of Ura4 has not been conducted in fission yeast. We therefore studied accumulation of a precursor of OMP in the *ura4* deletion mutant using mass spectrometry. We extracted metabolites according to the procedure described by Pluskal et al. [12]. The extract was separated by HPLC and absorbance at 260 nm was used to detect nucleic acids. A clear peak at 1.40 was detected when the *ura4* deletion mutant was grown on YPD media, which was not observed when WT cells were grown on YPD media (Fig. 7A; arrow head). This peak disappeared when the *ura4* deletion mutant was grown on uracil-containing medium (data not shown). Therefore this peak specifically appeared only in conditions in which cell lysis occurred. The peak did not merge with that of an OMP standard, ruling out the possibility that the peak was OMP (Fig. 7A; arrow).

Even though OMP was not detected by HPLC, mass spectrometric analysis in MRM mode identified the accumulation of OMP in the *ura4* deletion mutant when grown on YPD media, which was not observed in WT cells (Fig. 7B) or under conditions in which cell lysis did not occur, such as when Δ*ura4* cells were grown on YES media (data not shown). The product at 1.40 had the molecular weight (m/Z 288) of orotidine when analysed by TOF MS (Fig. 7C). Thus, the accumulation of low levels of OMP as well as a dephosphorylated form of OMP, orotidine, was identified in the *ura4* deletion mutant. Importantly, these products were only detected in samples of cells that had undergone cell lysis. A higher amount of uracil lowered the OMP production when the *ura4* deletion mutant was grown in YPD liquid medium (Fig. S2). We further tested the accumulation of these compounds in *ura3* deletion mutants of *S. cerevisiae*. Accumulation of orotidine was detected by HPLC in these mutants (Fig. 7D; arrow heads), but OMP was not detected by mass spectrometric analysis in MRM mode (Fig. 7E; arrows).

### Cell Lysis in *S. japonicus*

**Figure 8:**
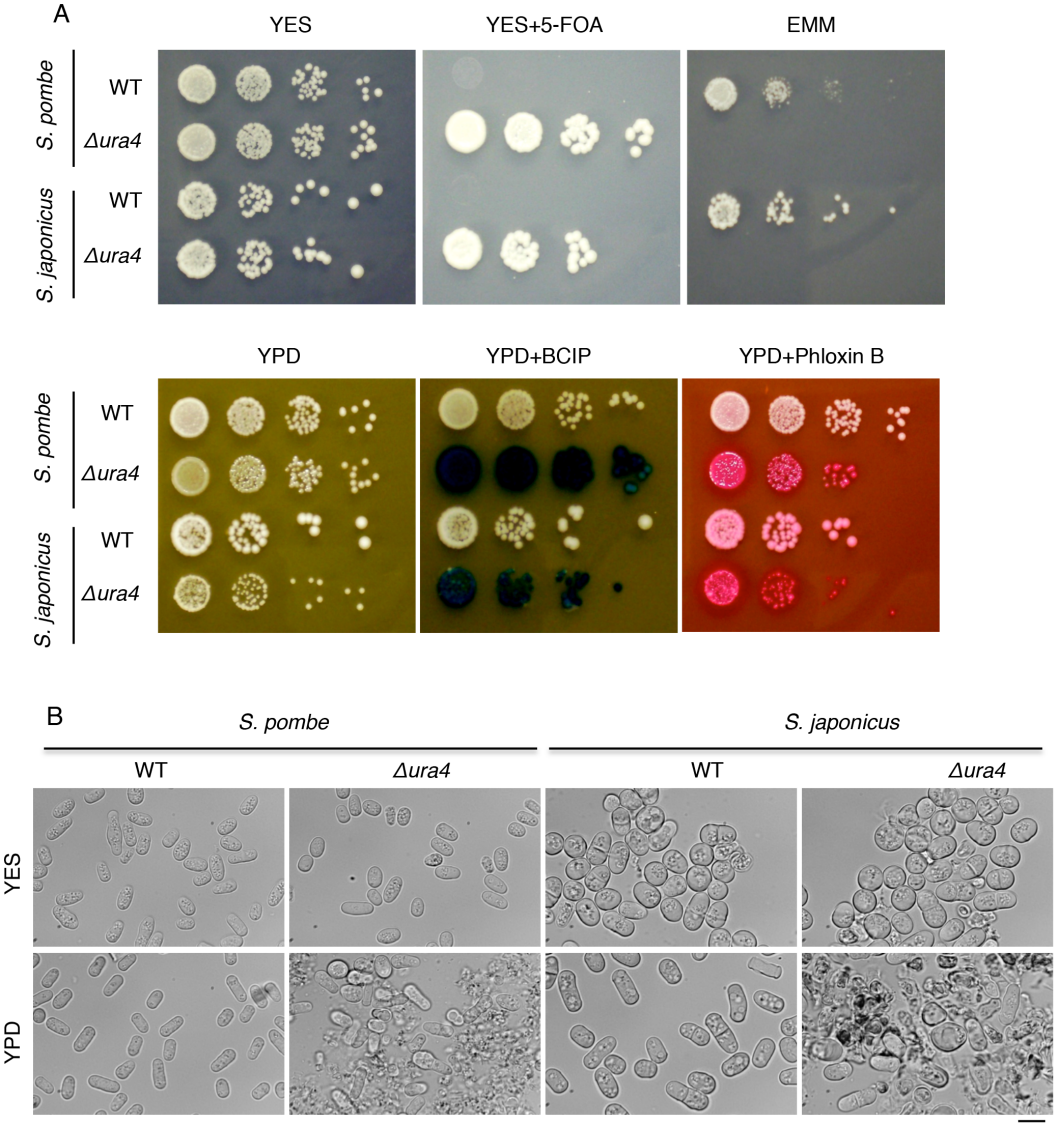
Cell lysis of the *S. japonicus ura4* deletion mutant in the presence of polypeptone. (A) Serially diluted cells (10-fold) of *S. pombe* L972 (WT) and UMP31 (Δ*ura4*), and *S. japonicus* NIG2028(*h^−^*) and NIG5091(*h^−^ ura4-D3*) strains were grown on the indicated plates and incubated for 3 days at 30°C. For the alkaline phosphatase assay, BCIP was used as described in Fig. 1. (B) Cellular morphologies of the indicated strains after being grown on YPD plates for 3 days at 30°C. Scale bar: 10 µm.

We next investigated whether cell lysis occurred in the *ura4* deletion mutant of *S. japonicas,* which was derived from the WT strain [24]. *S. japonicus* is a fission yeast that forms eight spores, and is closely related to *S. pombe.* BCIP and phloxin B staining (Fig. 8A) and cellular morphology (Fig. 8B) indicated that cells of the *S. japonicus ura4* deletion mutant underwent cell lysis when grown in the presence of polypeptone, whereas cells of the WT *S. japonicus* strain did not. This indicates that cell lysis of *ura4* deletion mutants grown in the presence of polypeptone is conserved in two fission yeast species.

## Discussion

We report the *ura4* deletion strains of *S. pombe* and *S. japonicus* undergo dramatic cell lysis when grown on media containing polypeptone or tryptone. The lysis phenotype was observed to some extent even when cells were grown in the commonly used YE medium, which contains a limited amount of adenine and uracil, and the phenotype was enhanced by the addition of polypeptone. The lysis phenotype of the *S. pombe ura4* deletion mutant was suppressed by the expression of *ura4* or *S. cerevisiae URA3*, or the addition of uracil to the media. The lysis phenotype is specific to *ura4* deletion mutant strains and was not observed in other *ura* deletion strains (*ura1*, *ura2*, *ura3*, and *ura5*). In addition, the cell lysis phenotype of the *ura4* deletion strains was suppressed by concomitant deletion of *ura1*, *ura2*, *ura3*, *ura5*, or *coq8,* which is required for Ura3 activity. These results indicate that the cell lysis phenotype was solely due to the deletion of *ura4*, and was not due to the general requirement of fission yeast for uracil. The lysis phenotype was not observed in the *S. cerevisiae URA3* deletion mutant, indicating that the cell lysis phenotype linked to *ura4* is specific to fission yeast.

The mutations of eight spontaneous revertants that overcame the cell lysis phenotype of the *ura4* deletion mutant resided in the *ura1* gene. It is reasonable that mutation of *ura1* would overcome the cell lysis phenotype of the *ura4* deletion mutant because Ura1 is upstream of Ura4 in UMP biosynthesis. However, it is unclear why all mutations in these revertants resided predominately in *ura1* rather than *ura2*, *ura3*, or *ura5*. The *ura1* gene (6 kb) is larger than the other *ura* genes, meaning that the rate of mutation in *ura1* may be higher than that of the other *ura* genes.

Cells of the *ura4* deletion strain dramatically lysed upon reaching stationary phase and this was suppressed by the addition of the osmotic stabiliser, sorbitol. The sensitivity of the *ura4* deletion strain to zymolyase and calcofluor white indicated that the cell wall integrity of this mutant is impaired. The cell wall of fission yeast is composed predominantly of polysaccharides, including (1,3) ß-D-glucan; (1,6) ß-D-glucan; (1,3) α-D-glucan [4]; galactomannan; and small amounts of chitin. Chitin is essential for *S. cerevisiae* growth but does not play an important role in mitotic growth of fission yeast [25], [26]. α-D-glucan is essential for the growth of fission yeast [4], [27], but not *S. cerevisiae*. Differences in the cell wall components of *S. pombe* and *S. cerevisiae* may explain why the cell lysis phenotype was not observed in the *S. cerevisiae URA3* deletion strain. If this is the case, α-glucan may be the cell wall component that is responsible for the perturbation of cell wall integrity in *ura4* deletion strains, as this component is absent from *S. cerevisiae*. Indeed, the main α-glucan synthase in *S. pombe* (Mok1/Ags1) is essential for growth and its temperature-sensitive mutant exhibits a cell lysis phenotype when grown at a restrictive temperature [27]. However, even if α-glucan is the target of *ura4*-mediated cell lysis, this does not explain why deletion of other *ura* genes apart from *ura4* did not cause cell lysis. If the level of uridine diphosphate (UDP)-glucose is solely responsible for α-glucan synthesis, cell lysis should be observed in all *ura* mutants.

Since the *ura4* gene encodes OMP decarboxylase that mediates the formation of UMP from OMP, *ura4* deletion strains were expected to accumulate OMP that might trigger cell lysis. Mass spectrometric analysis in MRM mode identified accumulation of OMP in the *ura4* mutant and HPLC identified an apparent peak of orotidine. Importantly, these products were only detected when cells were grown under conditions in which cell lysis occurred. Since OMP and cell lysis were not detected in *S. cerevisiae ura3* mutants when cells were grown on YPD, OMP may be a ‘cell lysis inducing factor’ in the *S. pombe ura4* deletion strains. One possibility is that OMP inhibits cell wall synthesis by preventing α-glucan synthesis from UDP-glucose and thereby inducing cell lysis. However, further study is required to test this hypothesis.

It is unknown which components of polypeptone enhance cell lysis in the *ura4* deletion strains. Casamino acids did not enhance the cell lysis phenotype. Since casamino acids are generated by complete hydrolysis of polypeptone, this suggests that amino acids do not trigger the cell lysis phenotype. As casein is converted to polypeptone by pepsin, specific peptides in polypeptone may trigger the cell lysis in the *ura4* deletion strains, although identifying such peptides will not be easy.

Because *ura4* is widely used as a selection marker in *S. pombe*, researchers need to be careful when interpreting phenotype of *ura4* mutants. The cell lysis phenotype is observed to some extent when *ura4* deletion mutants are grown in the commonly used YE medium, and this is enhanced by polypeptone. The phenotype should be compared to strains without any auxotrophic background.

## Supporting Information

Figure S1 **Cellular morphology of a tetrad, 5a (Δ** ***ura4*** **), 5b (** ***leu1-32*** **Δ** ***ura4*** **), 5c (** ***leu1-32*** **) and 5d (wild type), derived by crossing L972 (** ***h*** **^−^) with PR110 (** ***h*** **^+^** ***ura4-D18 leu1-32*** **).** Cells were grown on YES and YPD. Bar: 10 µm
(TIFF)

Figure S2 **(A) Cell’s culture of the L972 (WT), UMP34 (Δ** ***ura1*** **), UMP35 (Δ** ***ura2*** **), UMP36 (Δ** ***ura3*** **), UMP31 (Δ** ***ura4*** **), and UMP37 (Δ** ***ura5*** **) strains were serially diluted 10-fold, plated on the indicated plates and incubated for 3 days at 30°C.** For alkaline phosphatase assay, each plate was overlaid for 10, 30 and 60 min with a phosphatase assay solution as in Fig. 1. The amount of OMP was measured by mass spectrometry in UMP31 (Δ*ura4*) cells grown in YPD liquid medium containing an indicted amount of uracil. Under these conditions, a higher amount of uracil (900 mg/L) can only suppress the cell lysis phenotype.
(TIFF)

Table S1 **Oligonucleotide primers used in this study.**
(DOCX)
