# Correction: Polypeptone Induces Dramatic Cell Lysis in *ura4* Deletion Mutants of Fission Yeast

**DOI:** 10.1371/annotation/9c5955eb-06f7-46f9-aee8-a2f8952f6747

**Published:** 2013-08-23

**Authors:** Yuzy Matsuo, Kouhei Nishino, Kouhei Mizuno, Takashi Akihiro, Takashi Toda, Yasuhiro Matsuo, Tomohiro Kaino, Makoto Kawamukai

A funding organization for the fifth author was incorrectly omitted from the Funding Statement. The following sentence should be read after the first sentence of the Funding Statement: "TT is supported by Cancer Research UK." 

