# Supplementary figures and images for: Polypeptone Induces Dramatic Cell Lysis in ura4 Deletion Mutants of Fission Yeast
Source: PLoS One. 2013 Mar 21;8(3):e59887. doi: 10.1371/journal.pone.0059887 (PMC3605382; doi:10.1371/journal.pone.0059887)

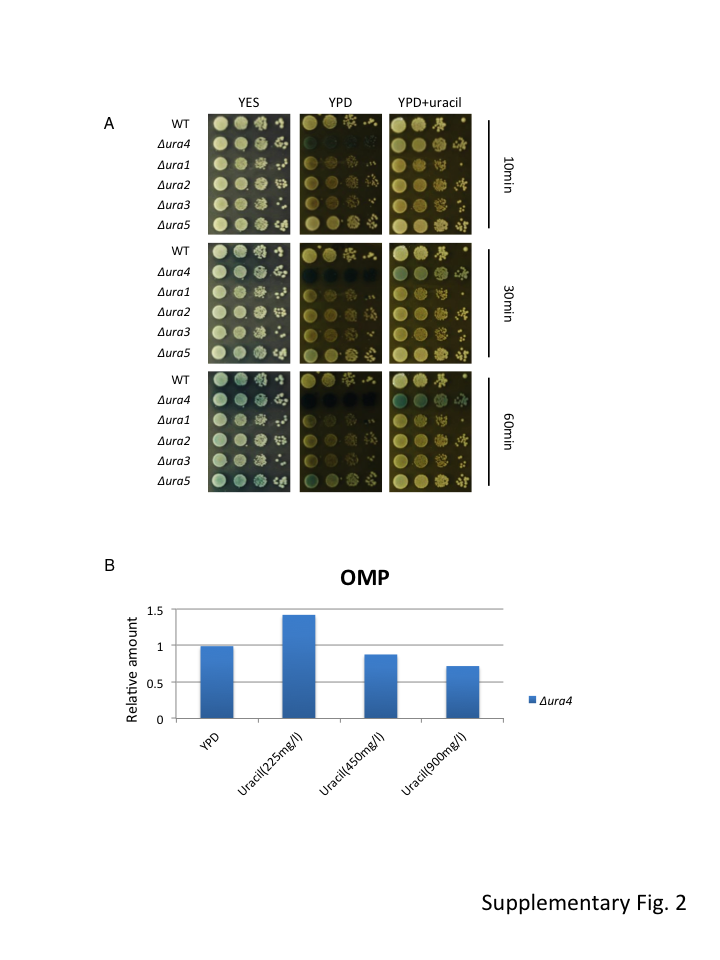

Supplement: Figure S2 — (A) Cell’s culture of the L972 (WT), UMP34 (Δ ura1 ), UMP35 (Δ ura2 ), UMP36 (Δ ura3 ), UMP31 (Δ ura4 ), and UMP37 (Δ ura5 ) strains were serially diluted 10-fold, plated on the indicated plates and incubated for 3 days at 30°C. For alkaline phosphatase assay, each plate was overlaid for 10, 30 and 60 min with a phosphatase assay solution as in Fig. 1. The amount of OMP was measured by mass spectrometry in UMP31 (Δura4) cells grown in YPD liquid medium containing an indicted amount of uracil. Under these conditions, a higher amount of uracil (900 mg/L) can only suppress the cell lysis phenotype. (TIFF) [file pone.0059887.s002.tiff]
