# Supplementary material for: Polypeptone Induces Dramatic Cell Lysis in ura4 Deletion Mutants of Fission Yeast
Source: PLoS One. 2013 Mar 21;8(3):e59887. doi: 10.1371/journal.pone.0059887 (PMC3605382; doi:10.1371/journal.pone.0059887)
Supplement: Table S1 — Oligonucleotide primers used in this study. (DOCX) [file pone.0059887.s003.docx]

| Table S1. Oligonucleotide primers used in this study. | |
| --- | --- |
| Primer name | Primer sequence (5’to 3’) |
| ura1 d1 F | gagctcaaactcattcactg |
| ura1 d2 R | cgtcgacctgcagcgtacgacacagtacttaggatatcgg |
| ura1 d3 F | cgagctcgaattcatcgatgagatactcagtgatgtgctg |
| ura1 d4 R | gggctgaatcctaccacaac |
| ura1 dis chk R | atttcgctaccaacaggtcg |
| ura2 d1 F | actgctttgtactgatgacc |
| ura2 d2 R | cgtcgacctgcagcgtacgaaggtagtgggtattgtcaag |
| ura2 d3 F | cgagctcgaattcatcgatgtgggagtaattcagtacatg |
| ura2 d4 R | tgtacgtaggttcttatacg |
| ura2 dis chk R | atcatgattaaggagctagg |
| ura3 d1 F | attgcctgtatcaacgaacg |
| ura3 d2 R | cgtcgacctgcagcgtacgaaggatcagaacagaatcgtc |
| ura3 d3 F | cgagctcgaattcatcgatgtcagtgtcgtttactggtac |
| ura3 d4 R | tgggaagttgagctctgaac |
| ura3 dis chk R | caaatgcctgcttgaaatcc |
| ura4 d1 F | tcttcctctagatgctcgtc |
| ura4 d2 R | cgtcgacctgcagcgtacgatgtgtcaaatctgacatggc |
| ura4 d3 F | cgagctcgaattcatcgatgtgctcctacaacattaccac |
| ura4 d4 R | gaagaacattactcgtcgtg |
| ura4 dis chk R | accaacatgcctgtaagtag |
| ura5 d1 F | gtggaagttaacgattgcac |
| ura5 d2 R | cgtcgacctgcagcgtacgatttctaagctctacatgggg |
| ura5 d3 F | cgagctcgaattcatcgatgggaggaaagagaataattgg |
| ura5 d4 R | ttatatgcgtctttcgaacg |
| ura5 dis chk R | agttaatcaccaggcttctg |
| coq8 d1 F | gtacgatagtaaaggtaaaaatg |
| coq8 d2 R | ggggatccgtcgacctgcagcgtacgaggaagggctttcaagtg |
| coq8 d3 F | gtttaaacgagctcgaattcatcgatcacattggttgaaaccct |
| coq8 d4 R | gaaagtctgcttcaaaggc |
| coq8 dis chk R | cgatagataaaggacatcac |
| pFA6a uniF | tcgtacgctgcaggtcgacg |
| pFA6a uni R | catcgatgaattcgagctcg |
| pFA6A uni chk F | gctaggatacagttctcaca |
| KanMX6 chk F | gaactgcctcggtgagtttt |
| MD1 | cggatccccgggttaattaaggcg |
| MD2 | gaattcgagctcgtttaaacactggatggcggcgttagtatcg |
| Pnmt1-ura1 | tagtcgctttgttaaatcatatgtccggattgctaccttc |
| ura1-Tnmt1 | caagggagacattccttttattaatttgctacttcagtag |
| Pnmt1-ura2 | tagtcgctttgttaaatcatatgagtttgaaaatacctgg |
| ura2-Tnmt1 | caagggagacattccttttattactcccatgaacagtgcc |
| Pnmt1-ura3 | tagtcgctttgttaaatcatatgtatcaacgatctttgtt |
| ura3-Tnmt1 | caagggagacattccttttactattcttccttgccaatta |
| Pnmt1-ura4 | tagtcgctttgttaaatcatatggatgctagagtatttcaaag |
| ura4-Tnmt1 | caagggagacattccttttaatgctgagaaagtctttgct |
| Pnmt1-ura5 | tagtcgctttgttaaatcatatgtcttataaacttgagtt |
| ura5-Tnmt1 | caagggagacattccttttatcatttggcttggtactgct |
| Pnmt1-URA3 | tagtcgctttgttaaatcatatgtcgaaagctacatataa |
| URA3-Tnmt1 | caagggagacattccttttattagttttgctggccgcatc |
| PTN-L1 T7 | taatacgactcactatagggcgaattggag |
| PTN-L1 T3 | aattaaccctcactaaagggaacaaaagct |
| Pnmt1 80bp F | ggcatatcatcaattgaata |
| Tnmt1 80bp R | taatatgcagcttgaatggg |
| ura1 Seq F1 | cgaaaagcggaagtgaaatg |
| ura1 Seq F2 | gtgtacattgtgctttgacg |
| ura1 Seq R | tatcttccaattccaaggcc |
